# Elevated carbon dioxide is predicted to promote coexistence among competing species in a trait‐based model

**DOI:** 10.1002/ece3.1733

**Published:** 2015-10-06

**Authors:** Ashehad A. Ali, Belinda E. Medlyn, Thomas G. Aubier, Kristine Y. Crous, Peter B. Reich

**Affiliations:** ^1^ Division of Earth and Environmental SciencesLos Alamos National LaboratoryLos AlamosNew Mexico USA; ^2^ Department of Civil and Environmental EngineeringUniversity of CaliforniaIrvineCaliforniaUSA; ^3^ Department of Biological SciencesFaculty of ScienceMacquarie UniversityNorth RydeNSW2109Australia; ^4^ Hawkesbury Institute for the EnvironmentUniversity of Western SydneyLocked Bag 1797PenrithNSW2751Australia; ^5^ UMR 5175Centre d'Ecologie Fonctionnelle et Evolutive1919 route de Mende 34090MontepellierFrance; ^6^ UMR 7205Muséum National d'Histoire NaturelleCP5045 rue Buffon75005ParisFrance; ^7^ Department of Forest ResourcesUniversity of MinnesotaSt. PaulMinnesotaUSA

**Keywords:** Elevated CO_2_, plant competition, species diversity, species traits

## Abstract

Differential species responses to atmospheric CO_2_ concentration (C_a_) could lead to quantitative changes in competition among species and community composition, with flow‐on effects for ecosystem function. However, there has been little theoretical analysis of how elevated C_a_ (eC_a_) will affect plant competition, or how composition of plant communities might change. Such theoretical analysis is needed for developing testable hypotheses to frame experimental research. Here, we investigated theoretically how plant competition might change under eC_a_ by implementing two alternative competition theories, resource use theory and resource capture theory, in a plant carbon and nitrogen cycling model. The model makes several novel predictions for the impact of eC_a_ on plant community composition. Using resource use theory, the model predicts that eC_a_ is unlikely to change species dominance in competition, but is likely to increase coexistence among species. Using resource capture theory, the model predicts that eC_a_ may increase community evenness. Collectively, both theories suggest that eC_a_ will favor coexistence and hence that species diversity should increase with eC_a_. Our theoretical analysis leads to a novel hypothesis for the impact of eC_a_ on plant community composition. This hypothesis has potential to help guide the design and interpretation of eC_a_ experiments.

## Introduction

Increases in atmospheric CO_2_ concentration (C_a_) have been shown to differentially affect plant species, with some species being more strongly responsive than others (Bazzaz 1990; Lloyd and Farquhar 1996; Poorter 1998). This difference among species in responsiveness to elevated C_a_ (eC_a_) could change the outcome of competitive interactions among plants (Bazzaz and McConnaughay 1992; Körner and Bazzaz 1996; Reynolds 1996; Brooker 2006), with cascading effects on the composition and diversity of plant communities (Zavaleta et al. 2003; Suding et al. 2005). However, there is currently relatively little theory predicting what kind of change in community composition should be expected, or what types of species should be favored under eC_a_.

Experimental work with young, individually grown plants shows that fast‐growing species typically benefit most from eC_a_ (Poorter and Navas 2003). One reason is that inherently fast‐growing species exhibit a greater absolute relative growth rate response to eC_a_ than their slow‐growing counterparts (Poorter 1993, 1998; Atkin et al. 1999). The difference can also be attributed in part to the greater allocation of biomass to leaf tissue and higher photosynthetic rates in fast‐growing species under eC_a_ (Oberbauer et al. 1985). In consequence, it is commonly assumed that community composition might shift toward fast‐growing (Körner and Bazzaz 1996) or weedy species (Bazzaz 1990) under eC_a_.

**Figure 1:**
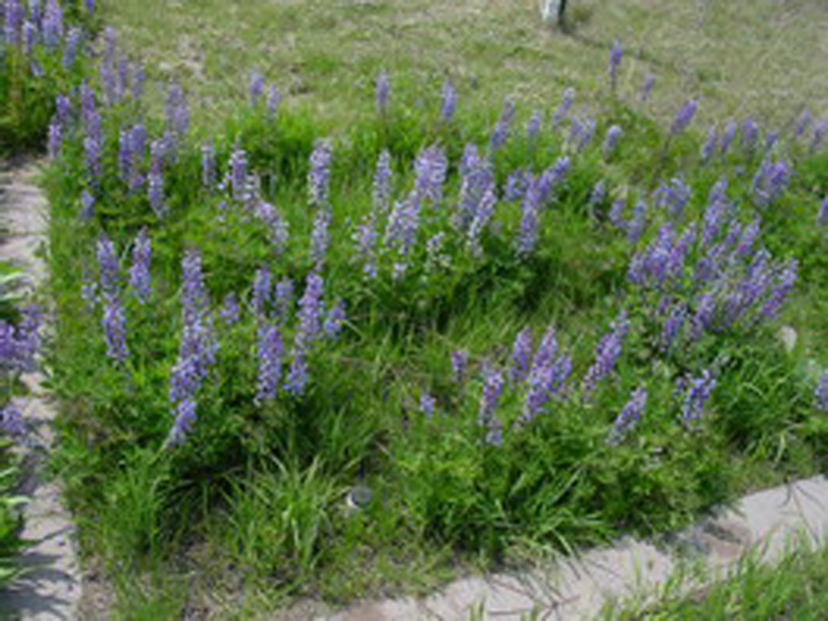
Mixtures of plant species in the BioCON FACE experiment at the University of Minnesota's Cedar Creek Ecosystem Science Reserve, MN, USA. Image courtesy of Kristine Y. Crous.

However, a recent study (Ali et al. 2013) showed on theoretical grounds that eC_a_ should only benefit fast‐growing species during the initial exponential growth phase; as plants reach canopy closure, slower‐growing plants should benefit the most (Ali et al. 2013). This theoretical prediction was supported by data from the Biodiversity × CO_2_ × N (BioCON, St Paul, MN, USA; Fig. 1) FACE experiment, in which relative biomass responses to eC_a_ were highest for the slowest‐growing species in plots where plants were grown in monocultures for 8 years. These results suggest that we need to rethink our predictions for how community composition may change under eC_a_ in field conditions. Given that experimental results appear to be somewhat mixed, with no consistent pattern emerging (Morgan et al. 2004), there is a clear need for theory‐based hypotheses against which to evaluate experimental data.

The goal of this study was to explore theoretically how community composition may change under eC_a_. As eC_a_ principally affects plant carbon uptake, with feedbacks via nitrogen availability (Comins and McMurtrie 1993), we examined competition between plants in terms of carbon and nutrient cycling. Similar frameworks to model competition have been widely used (Miki and Kondoh 2002; Rastetter and Agren 2002; Herbert et al. 2004; Daufresne and Hedin 2005; Ju and DeAngelis 2009). We simulated the effects of eC_a_ on long‐term outcomes of interspecific competition using the plant carbon–nitrogen model of Ali et al. (2013). This model represents a species as a vector of plant traits that determine carbon and nutrient uptake, such as photosynthetic nitrogen use efficiency and specific leaf area. By examining competition among species with different trait values, we aimed to identify which trait values would be most successful in competition under eC_a_, and whether communities would change in composition toward species with these trait values. Our ultimate goal was to generate testable hypotheses to guide experimental work.

As there is no consensus that a single mechanism of competition exists (Grime 1979; Chapin 1980; Tilman 1982; Thompson 1987; Huston and DeAngelis 1994; Hubbell 2001; Craine 2005; Craine et al. 2005), we implemented two alternative theories for competition in our model, so that our conclusions would not be contingent on the choice of the theory. Firstly, we implemented “resource use” theory (Tilman 1982), which predicts that the species that can reduce the monoculture soil nutrient availability to the lowest level (called R*) should, when grown in mixed‐species plots, eventually completely displace all other species if they are nutrient‐limited. The theory also predicts that the species that can reduce the incident light at the surface to lowest level (I*) should eventually displace all other species limited by light. There is some experimental evidence in support of this resource use theory (Tilman and Wedin 1991; Wedin and Tilman 1993; Huisman et al. 1999; Passarge et al. 2006; Dybzinski and Tilman 2007).

The second theory implemented is “resource capture” theory (Grime 1979), which hypothesizes that the outcome of competition is determined by the capacity of plants to capture and retain resources. A high rate of resource capture from the environment means “a high capacity for photosynthesis and nutrient uptake per unit tissue mass” (Chapin 1980). Herbert et al. (1999) proposed a theoretical framework based on this theory, whereby the model partitions resources captured between species according to their relative biomasses and rates of resource capture per unit biomass.

In this study, we implemented both competition theories (resource use and resource capture) in a plant C‐N model and investigated shifts in plant community composition and species richness under eC_a_. Two types of sensitivity analysis were made for both competition theories: one where species differed from each other in one trait value, and another where species differed in all trait values. Our goals were to determine, on theoretical grounds, whether eC_a_ is likely to change the outcome of plant competition; what types of species should emerge as successful competitors under eC_a_; and whether species richness is likely to be affected.

## Materials and Methods

### Plant production model

**Figure 2:**
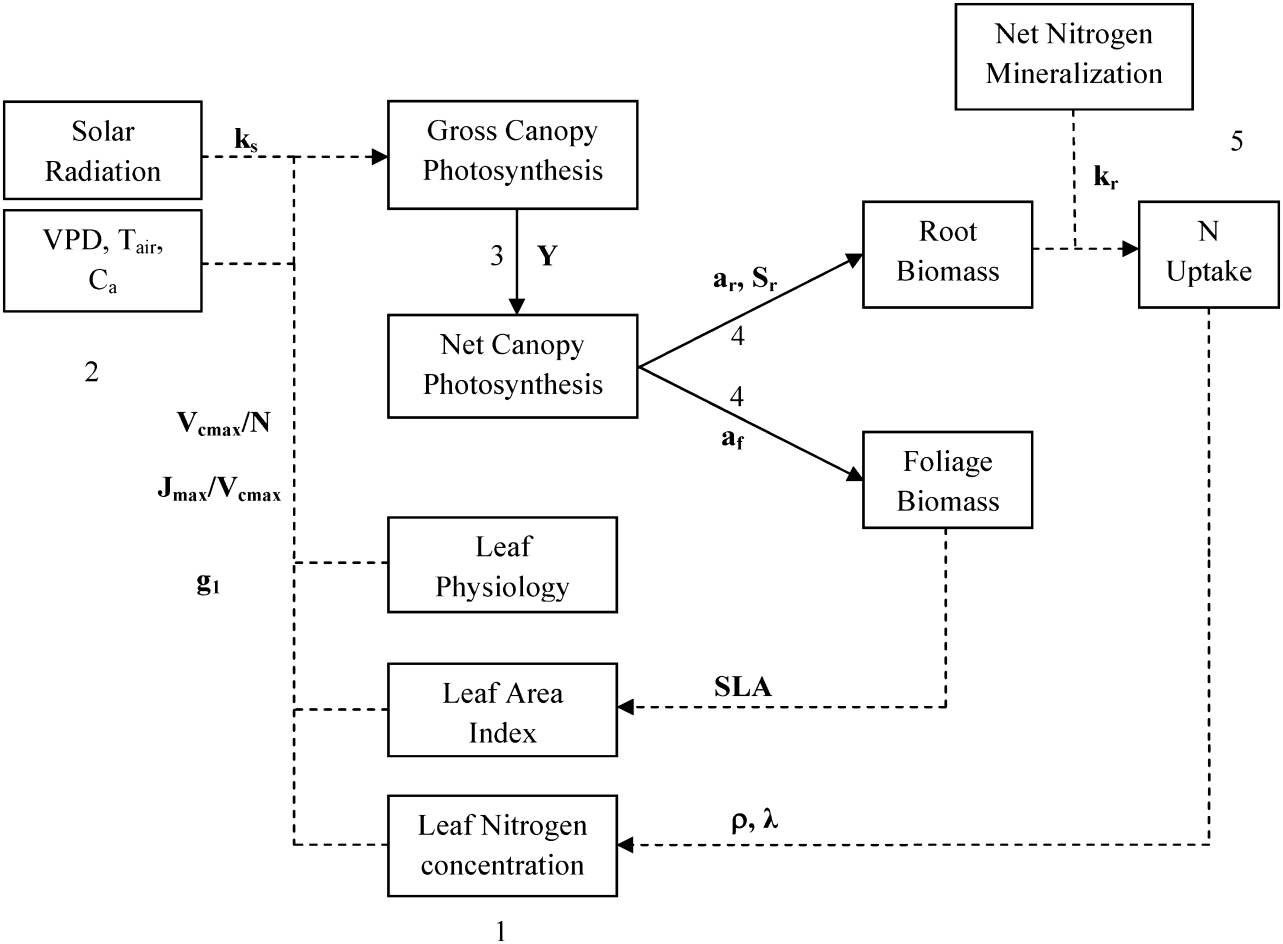
Flowchart of the model used in this study, showing how species traits (abbreviations in bold; defined in Table 1) are linked. Dashed lines are the flows of information (parameters, conversion, etc.), and solid lines are flows of carbon. Numbers indicate processes as follows: (1) scaling of leaf photosynthesis to the canopy, (2) meteorological data as driving variables, (3) subtraction of total respiration, (4) annual allocation of new biomass growth to plant compartments, namely foliage and roots, and (5) annual nitrogen uptake by the roots.

The simple plant production model used in this study (Fig. 2) was fully described in Ali et al. (2013). The model simulates plant nitrogen (N) and carbon (C) dynamics using a set of difference equations. The processes simulated include photosynthesis, respiration, carbon and nitrogen allocation, turnover, and nitrogen uptake. At the leaf scale, the response of photosynthesis to variations in light, temperature, and CO_2_ concentration is represented using the standard biochemical model of C_3_ photosynthesis (Farquhar and von Caemmerer 1982) and depends on the maximum Rubisco activity (*V*_*c*max_), which is a function of leaf nitrogen content. The leaf intercellular CO_2_ concentration, C_*i*_, is calculated from the optimal stomatal conductance model of Medlyn et al. (2011). Instantaneous leaf photosynthesis is calculated for sunlit and shaded leaf separately (Medlyn et al. 2000) using leaf area index (LAI, m^2^ m^−2^) and incident radiation. Daily canopy photosynthesis was calculated as the integral of the instantaneous photosynthesis. Whole‐plant respiration is assumed to be proportional to whole‐plant photosynthesis. Biomass increment of leaves and roots is a function of C allocation and turnover rates. N uptake is represented as a saturating function of root biomass (*B*_r_, g C m^−2^). For this study, the net soil N mineralization is held constant and equal to 3 g N m^−2^year^−1^. The model is deterministic, in common with other models examining grassland community dynamics (Parton et al. 1994; Cannell and Thornely 1998).

The model has twelve parameters that represent plant traits, which are listed in Table 1. In the model, a species is characterized as a vector of values for these plant trait parameters. Thus, growth rate of a range of different species can be simulated by varying the input parameters to the model.

**Table 1:** Species traits used in the model, together with units and values used in model simulations. Trait values were taken from the C_3_ grass and forb plant species at BioCON FACE experiment, Minnesota, USA (Table S1 (Ali et al. 2013)). Mean trait values across the species were used as baseline values in the simulations. For the sensitivity analysis, the range of trait values was obtained by varying each trait by ±50%

Trait	Definition	Baseline trait value [Range]	Units
*V*_*c*max_/N	Maximum leaf carboxylation rate per unit leaf nitrogen	52 [26,78]	*μ*mol g^−1^ N s^−1^
*J*_max_/*V*_*c*max_	Ratio of maximum electron transport to maximum carboxylation rate	1.86 [held constant]	Unitless
g_1_	Stomatal conductance operating point	3.7 [1.85,5.55]	kPa^0.5^
*Y*	Carbon use efficiency	0.5 [0.25,0.75]	Unitless
*k*_*s*_	Light extinction coefficient	0.6 [held constant]	m^2^ ground m^−2^ leaf
SLA	Specific leaf area	14 [7,21]	m^2^ leaf kg^−1^ DM
*a*_*f*_	Fraction of C allocated to leaves	0.4 [0.2,0.6]	Unitless
*a*_r_	Fraction of C allocated to roots (=1 – *a*_*f*_)	0.6 [0.8, 0.4]	Unitless
*S*_r_	Turnover rate of roots	0.75 [0.375,1.125]	per year
*k*_r_	Nitrogen uptake parameter related to root biomass	0.0239 [0.01195,0.03585]	m^2^ ground g^−1^ C
*ρ*	Ratio of root N: C to leaf N: C	0.6 [0.3,0.9]	Unitless
*λ*	Fraction of N retranslocated to the functioning foliage before senescence	0.5 [0.25,0.75]	Unitless

### Incorporating resource use theory

The idea behind resource use theory is that the species that depletes a limiting resource the most in monoculture (than any other) will be the winner of competition in a mixture. We consider two resources (light and nutrients) and focus on plants growing in conditions where these two resources are limiting.

The outcome of competition between two species (*A* and *B*) is determined by comparing their R* and I* values (Tilman 1997). The outcome is given as follows:

(1) $$\begin{array}{cc} & {R^{*}}_{A}<{R^{*}}_{B}\;\text{and}\;{I^{*}}_{A}<\;\;{I^{*}}_{B}\;\text{Species}\;A\;\text{wins}\\ & {R^{*}}_{A}>{R^{*}}_{B}\;\text{and}\;{I^{*}}_{A}>{I^{*}}_{B}\;\text{Species}\;B\;\text{wins}\\ & \left({R^{*}}_{A}>{R^{*}}_{B}\;\text{and}\;{I^{*}}_{A}<\;\;{I^{*}}_{B}\right)\;\text{or}\;\left({R^{*}}_{A}<{R^{*}}_{B}\;\text{and}\;{I^{*}}_{A}>{I^{*}}_{B}\right)\\ & \;\text{Both species coexist} \end{array}$$

That is, if either species has the lowest values for both R* and I*, that species wins; otherwise, both species can coexist.

The model has a well‐defined equilibrium point (NPP*) that can obtained by considering the carbon and nitrogen balances (see Appendix) (Ali et al. 2013). We calculated R* and I* values of each monoculture species at equilibrium as follows.

We calculated R* as the difference between annual net soil N mineralization rate, N_min_ (g N m^−2^ year^−1^), and the annual plant N uptake, N_up_ (g N m^−2^ year^−1^). N_min_ was held constant. Nitrogen uptake N_up_ was modeled as a saturating function of root biomass, *B*_r_ (g C m^−2^), and specific uptake rate, *k*_r_ (m^2^ g^−1^ C), which is analogous to the light extinction coefficient:

(2) $$N_{\mathrm{up}}=N_{\min}\;\left(1-\exp\left(-k_{r}B_{r}\right)\right)$$

The R* value of each species in monoculture was calculated at equilibrium. At equilibrium, the root biomass is related to equilibrium NPP by:

(3) $$B_{r}=\frac{1-a_{f}}{S_{r}}\text{NPP*}$$

where NPP* (g C m^−2^ year^−1^) is the equilibrium value of net primary production of the species, *S*_r_ (year^−1^) is the root turnover rate, and *a*_*f*_ (dimensionless) is the fraction of carbon allocated to foliage. Thus, R* is given by:

(4) $$R^{*}=N_{\min}\exp\left(-k_{r}\frac{1-a_{f}}{S_{r}}{\text{NPP}}^{*}\right)$$

Similarly to R*, I* was calculated as the difference between the annual incident photosynthetically active radiation (IPAR) (MJ m^−2^ year^−1^), which is constant, and the total amount of absorbed photosynthetically active radiation (APAR) (MJ m^−2^ year^−1^), which is a saturating function of the leaf area index (LAI, m^2^ m^−2^). At equilibrium, the leaf area index is given by:

(5) $$\text{LAI}=\frac{0.4*\text{SLA}a_{f}}{{\left[C\right]}_{f}}{\text{NPP}}^{*}$$

where SLA is the leaf area of the species (m^2^ leaf area kg^−1^ DM foliage biomass), 0.4 is a factor that scales specific leaf area to canopy‐level specific leaf area, and [*C*]_*f*_ is the foliage carbon concentration, taken to be 0.44 g C g^−1^ DM. Thus, I* is given by:

(6) $$I^{*}=\text{IPAR}\;\exp\;\left(-k_{s}\frac{0.4*\text{SLA}a_{f}}{{\left[C\right]}_{f}}{\text{NPP}}^{*}\right)$$

where *k*_*s*_ is the light extinction coefficient (m^2^ m^−2^). The values of R* and I* can then be compared among pairs of species to determine the outcome of competition between those species.

### Incorporating resource capture theory

In the resource capture theory, the amount of a resource captured by one species in competition depends on its biomass relative to that of competitors, as well its rate of resource capture per unit biomass. To implement this theory, the plant production model of Ali et al. (2013) was generalized to simulate the growth of two species growing in competition. Light and nutrients are the two limiting resources. The capture of these two resources between the competing species is calculated as a function of their relative biomass. We use equations presented by Herbert et al. (2004) to calculate the capture of these resources between the species, making the simplifying assumption that all species have equal canopy dominance, that is, no species is able to overtop another (*f*_*i*_ = 1 for all *i*, in Herbert et al. (2004)'s notation).

The total amount of PAR absorbed by both plant species is

(7) $${\text{APAR}}_{\mathrm{tot}}=\text{IPAR}\left(1-\exp\left(-k_{s1}{\text{LAI}}_{1}-k_{s2}{\text{LAI}}_{2}\right)\right)$$

where IPAR is the incoming irradiance (MJ m^−2^ year^−1^), *k*_*s*1_ and *k*_*s*2_ (m^−2^ m^2^) are the light extinction coefficients, and LAI_1_ and LAI_2_ are the leaf area indices of species 1 and 2, respectively. Following Herbert et al. (2004) and Ju and DeAngelis (2009), the fraction of this total radiation absorbed by species *i* in the presence of species *j* is given by:

(8) $$\frac{{\text{APAR}}_{i}}{{\text{APAR}}_{\mathrm{tot}}}=\frac{w_{C_{i}}}{w_{C_{i}}+w_{C_{j}}}$$

where the weighting factors $w_{C_{i}}$ are given by:

(9) $$w_{C_{i}}=\left(1-\exp\left(-k_{si}{\text{LAI}}_{i}\right)\right)\left(1+\exp\left(-k_{sj}{\text{LAI}}_{j}\right)\right)$$

The first factor in equation (8) represents the fraction of incident light that would be absorbed by species *i* in the absence of competition, while the second factor in equation (8) represents the competitive effect of species *i* on species *j* in absorbing light.

Combining equations (6), (7), (8), we obtain the amount of PAR absorbed by species *i* in competition with species *j*:

(10) $${\text{APAR}}_{i}=0.5\;\text{IPAR}\left(1-\exp\left(-k_{si}{\text{LAI}}_{i}-k_{sj}{\text{LAI}}_{j}\right)+\exp\left(-k_{sj}{\text{LAI}}_{j}\right)-\exp\left(-k_{si}{\text{LAI}}_{i}\right)\right)$$

The effect of equation (9) is that the fraction of total APAR partitioned to the species with the lowest light capture potential (*k*_*i*_ LAI_*i*_) is slightly greater than the ratio of the two species' light capture potentials (*k*_*i*_ LAI_*i*_/*k*_*j*_ LAI_*j*_). Where the light capture potentials are the same, the two species will absorb the same amount of light.

Net carbon production of species, NPP_*i*_, is then determined from the PAR absorbed by species *i* by multiplying it by a light use efficiency term that depends on leaf nitrogen concentration (Ali et al. 2013).

Competition for nutrients by root biomass is modeled in a very similar way. Total root nitrogen uptake for both species combined is given by:

(11) $$N_{\mathrm{up}}=N_{\min}\left(1-\exp\left(-k_{r1}B_{r1}-k_{r2}B_{r2}\right)\right)$$

where N_min_ is the net nitrogen mineralization (g N m^−2^ year^−1^), *k*_r1_ and *k*_r2_ (m^2^ g^−1^ C) are the root N uptake coefficients, and *B*_r1_ and *B*_r2_ (g C m^−2^) are the root biomass values for species 1 and 2, respectively. Note that in this model, N_min_ is assumed constant, that is, we ignore possible feedback effects via changing nitrogen mineralization rates. Following similar logic to the derivation for light capture, we obtain the root nitrogen uptake for species *i* as:

(12) $$\begin{array}{cc} N_{\mathrm{up},i} & =0.5\;N_{\min}(1-\exp\left(-k_{ri}B_{ri}-k_{rj}B_{rj}\right)\\ & +\exp\left(-k_{rj}B_{rj}\right)-\exp\left(-k_{ri}B_{ri}\right)) \end{array}$$

As with light capture, the outcome of this equation is that the fraction of total nitrogen uptake obtained by the species with the lowest nitrogen capture potential (*k*_*i*_ *B*_r*i*_) is slightly more than the ratio of the two species' nitrogen capture potentials (*k*_*i*_ *B*_r*i*_/*k*_*j*_ *B*_r*j*_).

The resource capture model with two species has a well‐defined equilibrium point (${\text{NPP}}_{i}^{*},\;{\text{NPP}}_{j}^{*}$) (see Appendix). Numerical simulation of the model with a daily time step was used to find this equilibrium point. The outcome of competition between two species is quantified by comparing the total biomass of the species. We defined the dominance ratio as the winning species' share of total biomass. This ratio ranges from 0.5 to 1. Both species are considered to coexist unless the biomass of one species vanishes to zero, in which case the dominance ratio = 1.

### Simulations

The simulation model was implemented as a discrete time‐step model in FORTRAN. Meteorological data were recycled each year. The model was run for 23 years, by which time the system was observed to have equilibrated. Simulated equilibrium points were verified against exact equilibrium values, calculated using the equations given in the Appendix, for a number of test cases.

Two types of simulations were run for both competition theories. Firstly, we examined the effects of individual trait values by considering competition between species which differed from each other in only one trait value. A set of species was generated by varying one trait at a time by ±50% of the base value and pairwise competition among this set of species was examined, using both resource use and resource capture theories. Secondly, we examined competition among species with trait values that were chosen from a random uniform distribution covering ±50% of the base value for each trait. For simplicity, we assumed that traits vary independently of one another; the effect of correlations among traits is considered in the Discussion. For resource use theory, we generated a set of 10,000 species and examined the outcome of competition among all possible pairs (10^8^ species pairs) of these species. Resource capture theory is more time‐intensive, so for this theory, we generated a second random set of 10,000 species and paired them with the first set of 10,000 species, thus generating 10,000 random species pairs, and examined competition between each pair. All model runs were carried out at ambient C_a_ (aC_a_, 360 ppm) and eC_a_ (550 ppm). Model simulations were carried out for aC_a_ at 360 ppm because it was near the level just prior to the start of the BioCON FACE experiment (Reich et al. 2001a,b; Crous et al. 2010).

## Results

### Competition among “species” differing in one trait value only

The results of the simulations where traits were varied singly are shown in Table 2. For resource use theory, species were ranked by assigning one point for each time the species won in paired competition, and 0.5 points for each time the outcome was coexistence. For resource capture theory, species were ranked by calculating their average fraction of total biomass in all possible pairwise competitions. While there are some differences in relative rankings of traits between resource use and resource capture theory, the two theories agree on the direction in which traits should change in order to increase success in competition. In both theories, the traits yielding the most success in competition were high fraction of carbon allocated to foliage (*a*_*f*_) and high carbon use efficiency (Y) (Table 2). The principal difference in trait rankings between theories was that slow root turnover (*S*_r_) promotes success in competition in resource capture theory, but has no effect on competition in resource use theory.

**Table 2:** Ranking of species traits by their effect on competitive ability under ambient and elevated C_a_. The rankings are shown for resource use and resource capture theories. Rankings were obtained by considering pairwise competition among a set of species differing by only one trait value. For resource use theory, each species was assigned 1 for each win, 0.5 for coexist, and 0 for each loss. For resource capture theory, the proportion of biomass obtained by the species in competition was averaged across the 17 pairwise competitions. High and low trait values are represented by “+,” “−,” respectively

Resource use			Resource capture		
Trait	Ability aC_a_	Ability eC_a_	Trait	Ability aC_a_	Ability eC_a_
+*a*_*f*_	17.0	16.0	+*a*_*f*_	0.82	0.80
+*Y*	15.5	16.0	+*Y*	0.81	0.78
+SLA	15.5	16.0	−*S*_r_	0.74	0.71
+*V*_*c*max_/N	13.5	13.5	+*V*_*c*max_/N	0.73	0.71
−*ρ*	13.5	13.5	−*ρ*	0.73	0.70
+*λ*	12.0	12.0	+SLA	0.71	0.70
+g_1_	9.5	9.0	+*λ*	0.66	0.64
−*S*_r_	9.0	9.0	+*k*_r_	0.63	0.60
+*S*_r_	9.0	9.0	+g_1_	0.60	0.59
+*k*_r_	9.0	9.0	−g_1_	0.50	0.51
−*k*_r_	8.5	9.0	−*λ*	0.49	0.50
−g_1_	5.5	5.5	+*ρ*	0.43	0.44
−*λ*	5.5	5.5	+*S*_r_	0.40	0.43
+*ρ*	4.0	4.0	−*k*_r_	0.27	0.35
−*V*_*c*max_/N	3.0	3.0	−SLA	0.20	0.23
−SLA	1.5	1.5	−*V*_*c*max_/N	0.19	0.22
−*Y*	1.5	1.5	−*a*_*f*_	0.07	0.07
−*a*_*f*_	0.0	0.0	−*Y*	0.02	0.03

Using resource use theory, the effect of eC_a_ on competition outcomes among pairs of species differing by single trait values was small, with the outcome of competition changing in only three species pairs (Table 2). In one of the three cases, a win–lose outcome became a coexistence outcome. As a result, the competitive ranking of species under eC_a_ barely changed.

Predicted values of NPP for two competing species differing in one trait value only using the resource capture theory are shown in Table 3, under aC_a_ and eC_a_. The outcome of competition in each case is shown by the dominance ratio, which is the dominant species' fraction of total productivity. In each case, the least productive species at aC_a_ is the most responsive to eC_a_. However, in no case does the least productive species at aC_a_ become the most productive species at eC_a_. Similarly, Table 2 demonstrates that the competitive rankings of species differing in only one trait value were unchanged between aC_a_ and eC_a_. However, the average dominance ratio of the top nine species decreased under eC_a_ and that of the bottom nine species increased. Thus, according to resource capture theory, eC_a_ does not alter which species dominates, but in each case competition becomes more even.

**Table 3:** Outcome of competition between two plant species differing in one trait, according to resource capture theory. One species had a high trait value while the other had a low trait value. The predicted values of net primary productivity (NPP) for each species at equilibrium under aC_a_ and eC_a_ are shown. The numbers in the brackets indicate the winning species dominance ratio. The enhancement ratios (E/A) are also shown for each species. Values for the winning species are indicated in bold

Traits	NPP (gC·m^−2^·year^−1^)		
	aC_a_	eC_a_	E/A ratio
*V*_*c*max_/N			
High	**348** (0.98)	**403** (0.93)	**1.16**
Low	7	22	3.34
g_1_			
High	**148** (0.63)	**176** (0.58)	**1.19**
Low	87	127	1.47
*Y*			
High	**457** (1.00)	**592** (1.00)	**1.30**
Low	0.05	0.21	4.53
SLA			
High	**294** (0.99)	**367** (0.98)	**1.24**
Low	3	8	2.84
*a*_*f*_			
High	**418** (1.00)	**530** (1.00)	**1.27**
Low	0.07	0.29	4.51
*S*_r_			
High	61	114	1.86
Low	**187** (0.75)	**199** (0.60)	**1.07**
*λ*			
High	**183** (0.71)	**216** (0.66)	**1.18**
Low	74	110	1.49
*k*_r_			
High	**236** (0.91)	**253** (0.78)	**1.07**
Low	24	71	2.91
*ρ*			
High	45	79	1.74
Low	**256** (0.85)	**287** (0.78)	**1.11**

### Competition among randomly generated species

We followed the simulations of competition between species differing in one trait only, with competition simulations among randomly generated species. Table 4 shows the mean trait values for the winning and losing species in pairs of randomly selected species under aC_a_ for both theories. For resource use theory, we compared the trait values of species that were outright winners of competition with those of the species that were losers in competition, whereas for resource capture theory, winners were classified as the species with a biomass share greater than 50%. The importance of each trait in determining the outcome of competition was evaluated by calculating the difference between the mean trait value of winners and the mean trait value of losers, divided by the mean trait value overall. Under resource use theory, the traits favoring success in competition were, in decreasing order of importance: high carbon use efficiency (*Y*), high fraction of carbon allocated to foliage (*a*_*f*_), high maximum leaf carboxylation rate per unit leaf nitrogen (*V*_*c*max_/N), high specific leaf area (SLA), high root nitrogen uptake parameter (*k*_r_), low root turnover rate (*S*_r_), low root to leaf nitrogen ratio (*ρ*), high fraction of nitrogen retranslocated to foliage (*λ*), and high stomatal conductance operating point (g_1_) (Table 4). Resource capture theory highlighted a similar set of traits as important in determining the outcome of competition; however, low *S*_r_ and low *ρ* were more important than high SLA and high *k*_r_.

**Table 4:** Comparison of mean trait values of winning and losing species when randomly generated species are compared for resource use and resource capture theories under aC_a_. Importance of each trait in determining the outcome of competition is calculated as the difference between the average winning and losing trait values, divided by the average trait value overall. Traits are ordered (in the descending order) by importance

Resource use theory				Resource capture theory			
Trait	Mean trait value, winning species	Mean trait value, losing species	Importance	Trait	Mean trait value, winning species	Mean trait value, losing species	Importance
*Y*	0.55	0.45	0.21	*Y*	0.55	0.46	0.17
*a*_*f*_	0.44	0.36	0.20	*a*_*f*_	0.43	0.37	0.14
*V*_*c*max_/N	55.17	48.35	0.13	*V*_*c*max_/N	54.71	49.22	0.11
SLA	14.78	13.19	0.11	*S*_r_	0.72	0.78	−0.08
*k*_r_	0.025	0.023	0.10	SLA	14.6	13.56	0.07
*S*_r_	0.71	0.79	−0.09	*ρ*	0.58	0.62	−0.06
*ρ*	0.58	0.63	−0.08	*k*_r_	0.025	0.023	0.05
*λ*	0.52	0.49	0.05	*λ*	0.51	0.49	0.04
g_1_	3.75	3.66	0.03	g_1_	3.74	3.67	0.02

**Figure 3:**
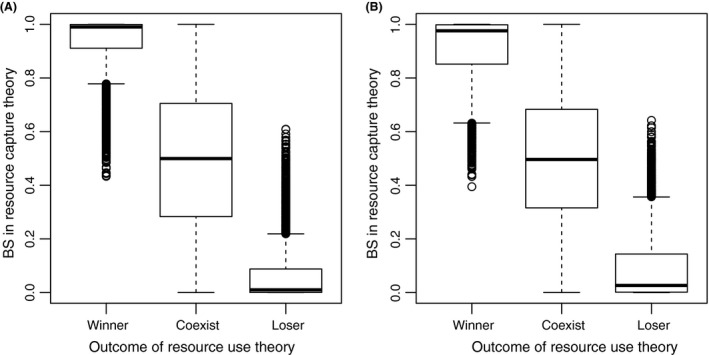
Comparison of the two competition theories. Both theories were applied to the same 10000 species pairs. The biomass share (BS) of each species predicted by resource capture theory is plotted against the outcome of resource use theory of the same species under aC_a_ (A) and eC_a_ (B).

To further compare the predictions for the outcome of competition by the two theories, we applied resource use theory to the 10,000 species pairs considered for resource capture and identified each species as winning, losing, or coexisting. Figure 3 shows the biomass share predicted by resource capture theory for species identified as winning, losing, or coexisting by resource use theory. Overall, the theories generally agree about the outcome of competition: “winners” in resource use theory almost always have a biomass share greater than 0.5 in resource capture theory, while “losers” almost always have a biomass share less than 0.5 (Fig. 3). “Coexisters” in resource use theory may have a biomass share anywhere from 0 to 1 in resource capture theory, but the values are centered on 0.5.

### Effect of eC_a_ on random species competition: Resource use theory

**Figure 4:**
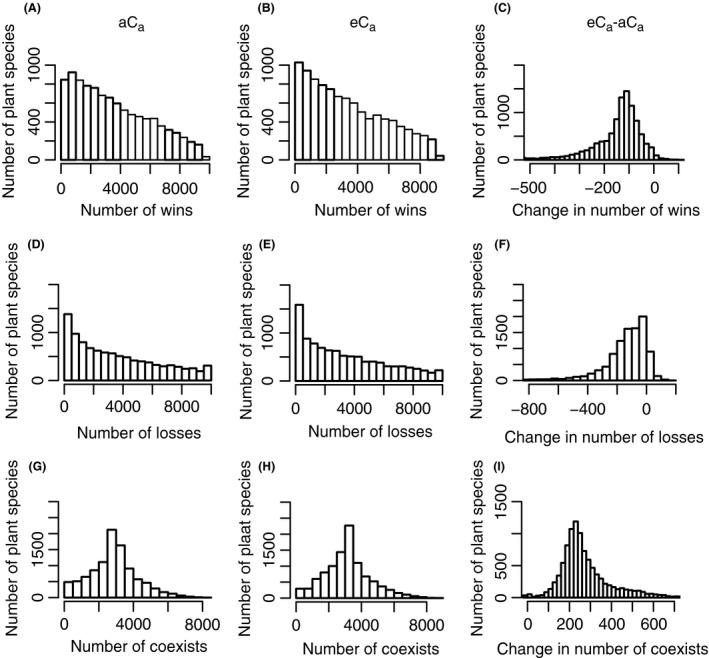
Outcome of pairwise competition among 10000 randomly generated species using resource use theory. For each species, the number of wins (A–C), losses (D–F), and coexistence cases (G–I) was recorded. The figures show histograms for the frequency of each of these outcomes, under (A, D, G) ambient and (B, E, H) elevated C_a_. The difference in the number of times that plant species was predicted to win, lose, or coexist under eC_a_ relative to aC_a_ (C, F, I) is also shown.

Using resource use theory, we calculated the frequency distributions of pairwise competition outcomes among 10,000 randomly generated species (Fig. 4). Competition outcomes were calculated for aC_a_ and eC_a_. For each species, the number of wins, losses, and coexistence cases was recorded. Under aC_a_, the frequency distributions of the number of wins and the number of losses are skewed to the right. Few species win often; most species win less than 3500 times of 9999. Similarly, only a few species lose often; most species lose less than 3500 times of 9999. However, the frequency distribution of coexistence is roughly normally distributed, with most species coexisting about 3500 times of 9999, and no species coexisting more than 8000 times.

Similar shaped distributions are found for eC_a_ (Fig. 4). However, eC_a_ caused some changes to the distributions. We investigated these by calculating, for each species, the difference in the number of times that plant species was predicted to win, lose, or coexist under changed environmental conditions relative to the aC_a_ case. The frequency distributions of these differences are shown in Figure 4. eC_a_ decreases the average number of wins (Fig. 4C) and average number of losses (Fig. 4F) but increases the average number of cases of coexistence (Fig. 4I). The interpretation is that increasing C_a_ tends to favor coexistence among species.

To identify whether eC_a_ favored any species traits in particular, we calculated the species' competitive ability by assigning 1 point for each win and 0.5 points for each coexistence. We then calculated the difference in competitive ability under ambient and eC_a_, and performed rank correlations to find which traits were most strongly associated with an increase in competitive ability (Table 5). An increase in competitive ability was strongly negatively associated with the fraction of carbon allocated to foliage (*a*_*f*_), the stomatal operating point (g_1_), and the maximum carboxylation rate per unit leaf nitrogen (*V*_*c*max_/N). These results indicate that the competitive ability of the species with low values of these traits is most strongly improved by growth under eC_a_. Interestingly, the increase in competitive ability with eC_a_ was not associated with carbon use efficiency (*Y*), despite the importance of this trait in determining competitive outcomes under aC_a_.

**Table 5:** Spearman's rank correlations of trait values with ambient NPP in monoculture; competitive ability at ambient C_a_ according to resource use theory; and with the change in competitive ability due to eC_a_. Traits are ordered (in the descending order) by the strength of correlation

Trait	Rank correlation with ambient NPP in monoculture	Trait	Rank correlation with ambient competitive ability	Trait	Rank correlation with change in competitive ability due to eC_a_
*Y*	0.60	*Y*	0.50	*a*_*f*_	−0.50
*a*_*f*_	0.51	*a*_*f*_	0.47	g_1_	−0.35
*V*_*c*max_/N	0.37	*V*_*c*max_/N	0.31	*V*_*c*max_/N	−0.27
*ρ*	−0.22	SLA	0.27	SLA	−0.18
*λ*	0.13	*k*_r_	0.24	*ρ*	0.15
SLA	0.10	*S*_r_	−0.23	*λ*	−0.11
g_1_	0.08	*ρ*	−0.19	*S*_r_	−0.10
*k*_r_	0.02	*λ*	0.11	*Y*	−0.08
*S*_r_	−0.01	g_1_	0.06	*k*_r_	0.06

### Effect of eC_a_ on random species competition: Resource capture theory

**Figure 5:**
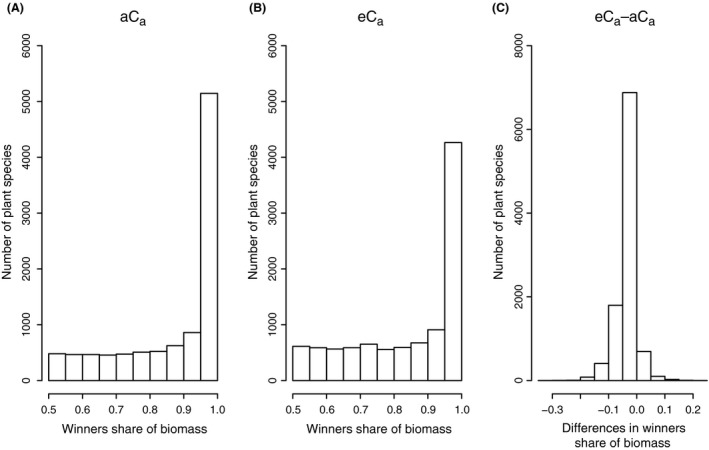
Outcome of competition in 10000 randomly generated pairs of species using resource capture theory. In each pair, the winner was identified and its share of biomass at equilibrium was calculated. The frequency distribution of the winners' share of biomass under (A) ambient and (B) elevated C_a_ is shown. The change in winners' share of biomass (C) is calculated as the share of biomass at eC_a_ less the share of biomass at aC_a_ for the winning species under aC_a_.

Using resource capture theory, we calculated the outcome of competition between 10,000 randomly generated pairs of species, at aC_a_ and eC_a_ (Fig. 5). For each species pair, we identified the winning species as that with the highest equilibrium biomass at aC_a_ and calculated the winner's share of biomass at aC_a_ and eC_a_, and the difference between the two. The frequency distributions of these numbers are shown in Figure 5.

**Figure 6:**
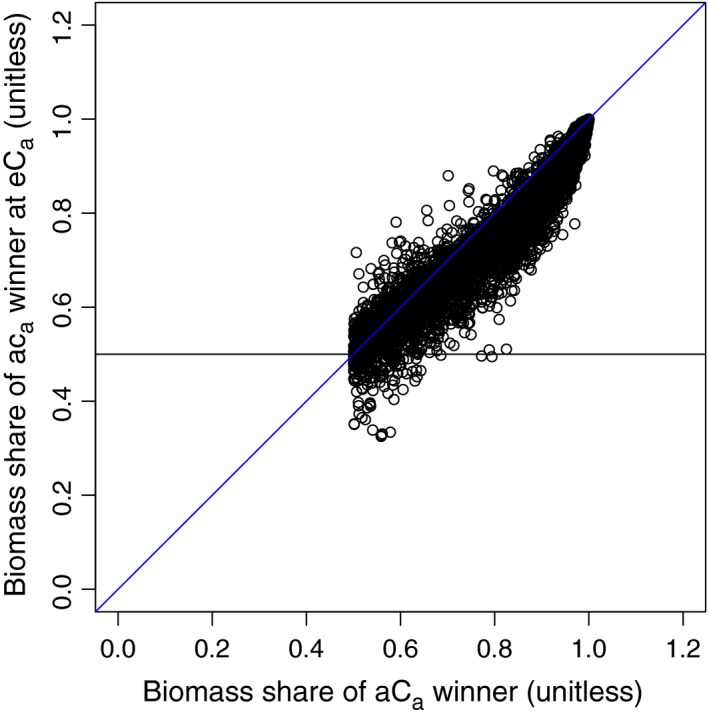
Biomass share (BS) of the ambient winner at eC_a_ as a function of its BS at aC_a_. The horizontal line indicates where the biomass share of the ambient winner at eC_a_ is 0.5. Points falling below this line indicate species pairs where the dominant species changed between aCa and eCa. The blue line is the 1:1 line. Points above this line indicate species pairs where the dominance ratio of the aC_a_ winner was increased at eC_a_.

Under aC_a_, the frequency distribution of the winners' share of biomass is skewed to the left, indicating that in most species pairs, the winner has over 90% of the total biomass. Under eC_a_, the frequency distribution is less skewed, indicating that the number of species with high biomass share is decreasing. In most cases, the winner's share of biomass under eC_a_ is lower than under aC_a_ (Fig. 6). However, it is rare for eC_a_ to change which species has the largest biomass share; this occurred in only 192 of 10,000 cases. The implication is that, although eC_a_ does not change the outcome of competition, it tends to make the competition more even.

## Discussion

Two approaches to modeling competition give consistent results – that eC_a_ tends to lessen the difference in competitive differences between species and can therefore increase coexistence. Our model predicts that more species will coexist and biomass share will be more even (less likely to be one very dominant species) but does not predict that winners will change. Our model predicts reduced competition in eC_a_ and increased species richness. This work provides a novel, mechanistic hypothesis for the outcomes of competition under eC_a_ that can be tested experimentally. Importantly, using a mathematical model to develop the hypothesis, the assumptions and logic underpinning the hypothesis are explicit, meaning that not only the overall prediction but also the underlying mechanisms can be tested against data.

The reason the model predicts increased evenness and increased diversity stems from the original prediction that eC_a_ will increase productivity in slow‐growing plants relatively more than in fast‐growing plants (Ali et al. 2013). In the resource capture theory, this results in the less dominant species being favored by eC_a_, reducing the dominance ratio (Table 3). The prediction of increased coexistence using resource use theory can be understood as follows. The condition for coexistence (Eq 1) can be rewritten using equations (4) and (6) as:

(13) $$\mathrm{or}\begin{array}{ccc} \frac{a_{fA{\mathrm{SLA}}_{A}k_{sA}}}{a_{fB{\mathrm{SLA}}_{B}k_{sB}}}<\frac{{\mathrm{NPP}}_{B}}{{\mathrm{NPP}}_{A}}<\frac{a_{rA}s_{rA}k_{rA}}{a_{rB}s_{rB}k_{rB}} & \;\;\;\mathrm{if} & \;\;\;\;\;\;\frac{a_{fA{\mathrm{SLA}}_{A}k_{sA}}}{a_{fB{\mathrm{SLA}}_{B}k_{sB}}}<\frac{a_{rA}s_{rA}k_{rA}}{a_{rB}s_{rB}k_{rB}}\\ \frac{a_{fA{\mathrm{SLA}}_{A}k_{sA}}}{a_{fB{\mathrm{SLA}}_{B}k_{sB}}}>\frac{{\mathrm{NPP}}_{B}}{{\mathrm{NPP}}_{A}}>\frac{a_{rA}s_{rA}k_{rA}}{a_{rB}s_{rB}k_{rB}} & \;\;\;\;\;\mathrm{if}\;\;\; & \frac{a_{fA{\mathrm{SLA}}_{A}k_{sA}}}{a_{fB{\mathrm{SLA}}_{B}k_{sB}}}>\frac{a_{rA}s_{rA}k_{rA}}{a_{rB}s_{rB}k_{rB}} \end{array}$$

where subscripts *A* and *B* indicate species *A* and *B*. That is, coexistence occurs when the ratio of NPP of the two species grown in monoculture falls within upper and lower bounds set by their relative trait values. As eC_a_ increases NPP of slow‐growing species by more, the ratio NPP_*B*_/NPP_*A*_ has fewer extreme values under eC_a_, with the implication that it will fall more often between these bounds, making coexistence more likely.

### Which trait values are favored by eC_a_?

We also examined which species traits are most strongly associated with the outcome of competition under aC_a_, and which traits are associated with improved competitive status under eC_a_. Although we have generalized the results to talk about slow‐growing vs fast‐growing species, there are some distinctions among the plant traits causing slow growth. Three traits, low foliage allocation (*a*_*f*_), low photosynthetic nitrogen use efficiency (*V*_*c*max_/N), and low carbon use efficiency (*Y*), are associated with low NPP at aC_a_. However, only two of these traits, low *a*_*f*_ and low *V*_*c*max_/N, are strongly associated with increased competitive ability under eC_a_. In contrast, the stomatal operating point g_1_ is only weakly associated with NPP at aC_a_, but was strongly associated with increased competitive ability under eC_a_. This result is consistent with the conclusions of Ali et al. (2013) who found that the trait g_1_ was important in determining the relative plant response to eC_a_. Thus, we suggest that experiments investigating competition under eC_a_ should also aim to quantify species traits, as the traits themselves, rather than growth rates per se, can be important in determining the effect of eC_a_ on competitive ability.

When ranking the importance of the traits, it is also important to consider the range of actual trait values among the species considered. When we ran our competition model for the seven species growing in the BioCON experiment, we did not find that the trait g_1_ was important in determining competition outcomes, simply because the values of g_1_ were very similar among this set of species (Ali 2012).

### Comparison with alternative hypotheses for plant competition outcomes under eC_a_

One existing hypothesis for the effects of C_a_ on plant community composition is that weedy and fast‐growing species may be favored, promoting invasions (Bazzaz 1990). Our model predictions differ strongly from this hypothesis; the model suggests that the dominance of fast‐growing species will be reduced under eC_a_ compared to aC_a_. The difference between these hypotheses arises from the time‐scale considered: the observation that fast‐growing species are more strongly responsive to eC_a_ derives from short‐term pot experiments, whereas our model applies to longer‐term field experiments (Ali et al. 2013). Our model prediction agrees with Dukes (2002) who demonstrates that the response of invasive species to eC_a_ in the field cannot be predicted from the response in a short‐term glasshouse experiment.

A related hypothesis is the idea that eC_a_ can drive competitive exclusion. Elevated C_a_ increases ecosystem productivity (Oren et al. 2001; Ainsworth and Long 2005; Reich et al. 2006a,b) which could potentially lead to competitive exclusion and decreasing diversity (Bazzaz and Garbutt 1988; Potvin and Vasseur 1997; Körner 2003; Brooker 2006; Lau et al. 2010). Our model does not yield this result because elevated C_a_ is predicted to increase productivity in both strong and weak competitors. Other authors have suggested that higher productivity with eC_a_ should increase diversity, based on a large‐scale empirical relationship between diversity and productivity (Woodward and Kelly 2008). Our model differs from this work because it does not assume a relationship between diversity and productivity; rather, we predict the outcome of competition based on underlying ecophysiological mechanisms.

Other hypotheses relate to the interaction between C_a_ and nutrient availability. Berry and Roderick (2002) suggested that in low nutrient environments, nutrient efficient species, such as sclerophylls, might respond more to eC_a_ than nutrient inefficient species and hence might increase in dominance. Our model does not yield this result because it predicts that nutrient inefficient species (those with low *V*_*c*max_/N) should actually respond more to eC_a_ under nutrient limitation, than nutrient efficient species (Ali et al. 2013). This prediction comes from the fact that (in the model) the nutrient inefficient species have low productivity at low nutrient availability and can thus benefit more strongly from the increased carbon availability under eC_a_. This prediction could also be tested experimentally.

Alternatively, it can be argued that the change in plant stoichiometry due to eC_a_ (Ainsworth and Long 2005; Novotny et al. 2007) could result in greater relative limitations by other dominant resources such as nitrogen (Reich et al. 2006a,b), and this effect should reduce competitive exclusion and increase species richness. Our model predictions are closest to this hypothesis, although our logic is subtly different. We assume that nitrogen is always limiting to plant growth, and the reduction in competitive exclusion arises from the fact that productivity of different plant species is more similar under eC_a_ than aC_a,_ and therefore, one species cannot outcompete another so readily.

### Comparison of the model with experimental data

The purpose of our model was to provide a logical theoretical framework with which to examine the results of experiments on the effect of eC_a_ on plant competition. The model is based on a set of simple but defensible assumptions. If it fails to predict experimental outcomes, it should be possible to identify which assumptions are at fault, and thereby increase our understanding of plant competitive relationships under eC_a_.

It is important to be aware of the assumptions made when testing the model against experimental data. We assumed that the vegetation is perennial, herbaceous, C_3_ and not leguminous, and that light and nitrogen availability are limiting to growth but water availability is not. As the predictions are made for the equilibrium situation, the model predictions are applicable to longer‐term ecosystem‐scale experiments with steady‐state plant canopies, rather than short‐term experiments in which canopies are still expanding. We also assumed that the canopy dominance factor (Herbert et al. 2004) was equal to one, implying that all species have similar height and rooting depth.

Although a number of experiments show results that contradict our model predictions, this may be because these model assumptions do not hold for these experiments. For example, Zavaleta et al. (2003) examined plant diversity responses in California annual grassland to eC_a_ and found reduced plant diversity after 3 years. However, this site experiences strong water limitation, and one reason for the reduction in plant diversity is that eC_a_, by relieving water stress, can delay senescence of the dominant plant canopy at the end of the growing season, narrowing the window when sufficient light would be available for the late‐emerging species. Many competitive interactions are driven by water availability, and it is clear that there is an important role for water limitations in determining competitive outcomes under eC_a_ (e.g., Polley et al. 1997). Our model must be seen as limited because it does not consider such interactions; there is an urgent need for theoretical studies extending our work to consider water‐limited environments.

A number of experimental findings on non‐water‐stressed C_3_‐dominated herbaceous communities do provide support for our model prediction that eC_a_ will increase evenness and species richness, although it should be acknowledged that these experiments also include C_4_ and leguminous species. In a long‐term field study on biodiversity of grasslands under eC_a_ conditions, community evenness was increased (Leadley et al. 1999), that is, dominance was reduced, in agreement with our theory. At the BioCON FACE experiment in Minnesota, eC_a_ partially eliminated negative effects on diversity of elevated N supply by reducing competitive exclusion (Reich 2009), and overall tended to increase plant diversity (Isbell et al. 2013). In the New Zealand grassland FACE experiment, productivity of the dominant grasses was not increased under eC_a_ but productivity of the subdominant forbs was increased (Newton et al. 2006). In a mixed‐grass prairie experiment, community evenness was found to increase with eC_a_ due to decreases in biomass of the dominant species (Zelikova et al. 2014). The support provided by these experiments for our theory is clearly insufficient as a formal test of the model, but does demonstrate that our model predictions deserve further experimental exploration.

### Model limitations and further work

Our model is intentionally simple, to enable its behavior to be readily understood. As a result, however, a number of other processes that are potentially important in determining the outcome of interspecific competition are missing from our model. We mentioned the need to extend the model to consider water limitation above. Additionally, our model does not consider population‐level processes, such as allocation of biomass to reproduction, recruitment, and mortality (Moorcroft et al. 2001). Neither resource use theory nor resource partitioning theory take account of these processes, so the model would need to be significantly extended to incorporate these population processes. In addition, experimental data to parameterize and test the effect of eC_a_ on these processes are as yet rather limited. Thus, there is considerable work to be done to add these effects into our model.

Our model does not consider the possible role of preemptive resource capture. We did not consider alternative timings for leaf area dynamics, for example. Similarly, when applying resource partitioning theory, we assumed that the canopy dominance factor, which takes into account the relative height of the two species, is zero, meaning that the species are equal in height. Similarly, the soil dominance factor was also assumed to be zero. Further work could consider how preemptive resource capture, either in time or space, may change the outcome of competition under eC_a_.

In this work, we assumed that species traits could vary independently from each other. In nature, there are significant correlations among some traits, such as leaf longevity and specific leaf area (Wright et al. 2004). To explore such trait correlations, we used our current model and made some additional analyses that included implementation of the leaf economics spectrum (Wright et al. 2004). We generated additional sets of random species, where we constrained some traits by implementing linear relationships between *S*_*f*_ and SLA, and among the traits *Y*,* V*_*c*max_/N, and *k*_r_ (Aubier 2013). These results are not shown here because we found little impact on the outcomes of the model, indicating that our current model predictions are robust to the implementation of trait correlations (Wright et al. 2004).

Our model only considers competition between two species, whereas most grasslands consist of many more than two species coexisting. Resource use theory assumes that the number of resources available determines the number of potentially coexisting species. Hence, we could not extend this theory to consider more species without also considering additional resources. However, resource partitioning theory allows for many species to coexist even though they are competing for a limited number of resources (Rastetter and Ågren 2002). Thus, our resource partitioning model could be fairly readily extended to consider more than two competing species. We consider it highly likely that our main finding in this study, that eC_a_ promotes coexistence, would continue to hold in a model of more than two species.

## Conclusion

We applied resource use theory to a plant carbon–nitrogen model in order to develop theory for how eC_a_ is likely to change competition among plant species. Use of the model allowed us to develop several testable hypotheses that we suggest could be examined in field experiments to enhance our understanding of competitive relations under eC_a_. Firstly, we identified the species traits increasing success in competition. In all analyses, the traits of high foliage allocation, high carbon use efficiency, and high photosynthetic nitrogen use efficiency led to strong performance in competition. These rankings among species traits could be used to examine outcomes of field‐based competition experiments to test whether species performance in competition can be predicted by their trait combinations.

Secondly, our model makes the novel prediction that eC_a_ is likely to make competition among species more even, with fewer strongly dominant species. With resource use theory, we predicted increased coexistence, implying increased diversity. With resource capture theory, we predicted that eC_a_ would reduce the dominance ratio of the winning species, increasing community evenness. These predictions could form a framework for studies of eC_a_ effects on competition in the field.

### Code availability

The present code is written in FORTRAN programming language. It uses R software for generating large set of species and MathCAD software for pairwise comparisons. It can also be obtained upon request by sending an email to ali.ashehad@gmail.com.

## Conflict of Interest

None declared.

## Figures and Tables

**Figure A1:**
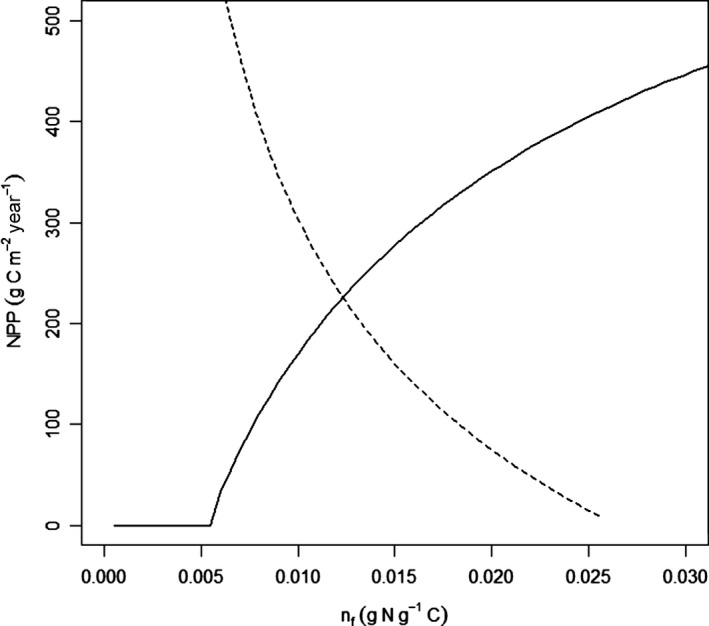
Equilibrium NPP for one species growing in monoculture is given by intersection of production constraint (solid line) and nutrient availability constraint (dashed line).

**Figure A2:**
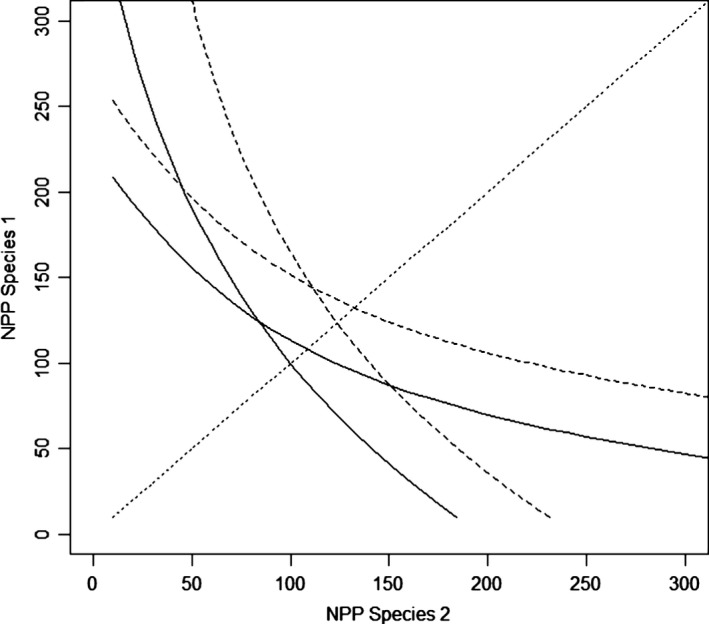
Graphical representation of equilibrium solution for two species growing in competition using resource partitioning model. The system equilibrium is given by the intersection of two constraint curves describing equilibrium NPP for one species as a function of the NPP of the other species. Solid lines, constraint curves at ambient C_a_; dashed lines, constraint curves at elevated C_a._ In this example, the species differ in the parameter *a*_*f*_.

## Equilibrium analysis of model

### Single species model

We follow the approach used by Comins and McMurtrie (1993) to derive the equilibrium values of the model. In this approach, equilibrium NPP (NPP*) is calculated by considering C and N balances of the plant.

In our simple, single species model, the N balance constraint is obtained by considering plant N balance. In equilibrium, N uptake by roots must equal N used in new growth. Hence,

(A1) $$N_{\min}\left(1-\exp\left(-k_{r}B_{r}^{*}\right)\right)={\text{NPP}}^{*}\left(a_{f}n_{f}^{*}+\left(1-a_{f}\right)\rho n_{f}^{*}\right)$$

where $n_{f}^{*}$ is foliage nitrogen concentration at equilibrium. Also at equilibrium, growth of roots must equal senescence. Thus,

(A2) $$B_{r}^{*}={\text{NPP}}^{*}\left(1-a_{f}\right)/s_{r}$$

Combining equations (A1) and (A2) yields an implicit equation for NPP* as a function of $n_{f}^{*}$

(A3) $$N_{\min}\left(1-\exp\left(-k_{r}{\text{NPP}}^{*}\left(1-a_{f}\right)/s_{r}\right)={\text{NPP}}^{*}\left(a_{f}n_{f}^{*}+\left(1-a_{f}\right)\rho n_{f}^{*}\right)\right)$$

The plant C balance constraint is obtained by considering the plant photosynthetic uptake. We use a simplified plant photosynthesis model here to demonstrate the equilibrium approach. We assume a light‐use efficiency expression

(A4) $$\text{NPP}=I_{0}\varepsilon \left(n_{f},C_{a}\right)\left(1-\exp\left(-k_{s}\text{LAI}\right)\right)Y$$

where *I*_0_ is annual incident radiation, the term (1 – exp(−k_*s*_ LAI)) yields the fraction of radiation absorbed, and *ε*(*n*_*f*_, C_a_) is the light‐use efficiency, which depends on the plant traits *V*_*c*max_/N and g_1._

At equilibrium, leaf area index is given by

(A5) $${\text{LAI}}^{*}={\text{NPP}}^{*}a_{f}*0.4*\text{SLA}/\text{Cfrac}$$

Combining eqs (A4) and (A5) enables us to obtain a second implicit equation for NPP* as a function of $n_{f}^{*}$:

(A6) $${\text{NPP}}^{*}=I_{0}\varepsilon \left(n_{f},C_{a}\right)\left(1-\exp\left(-k_{s}{\text{NPP}}^{*}a_{f}*0.4*\text{SLA}/\text{Cfrac}\right)\right)Y$$

The equilibrium NPP is given by the intersection of the two constraints (A3) and (A6), as shown in Figure A1. Alternatively, we can rearrange equation (A3) to obtain $n_{f}^{*}$ as

(A7) $$\begin{array}{cc} n_{f}^{*}= & \;\;N_{\min}\left(1-\exp\left(-k_{r}{\text{NPP}}^{*}\left(1-a_{f}\right)/s_{r}\right)\right)/\\ & \left({\text{NPP}}^{*}\left(a_{f}+\left(1-a_{f}\right)\rho \right)\right) \end{array}$$

and substitute this expression into (A6), to obtain a single equation that yields NPP*.

### Two species competing

With two species, we need to find equilibrium NPP for each species, given by ${\text{NPP}}_{i}^{*}$ and ${\text{NPP}}_{j}^{*}$. We can generalize the above analysis as follows.

Firstly, from equation (10) we have

(A8) $${\mathrm{APAR}}_{i}=0.5\;I_{0}\left(1-\exp\left(-k_{si}{\text{LAI}}_{i}-k_{sj}{\text{LAI}}_{j}\right)+\exp\left(-k_{sj}{\text{LAI}}_{j}\right)-\exp\left(-k_{si}{\text{LAI}}_{i}\right)\right)$$

And from equation (12) we have

(A9) $$\begin{array}{cc} N_{\mathrm{up},i}= & \;\;0.5N_{\min}(1-\exp\left(-k_{ri}B_{ri}-k_{rj}B_{rj}\right)\\ & +\exp\left(-k_{rj}B_{rj}\right)-\exp\left(-k_{ri}B_{ri}\right)) \end{array}$$

These equations can replace their simpler single‐species versions in equations (A1) and (A4). Equilibrium values of $B_{ri}^{*},\;B_{rj}^{*},\;{\text{LAI}}_{i}^{*}$, and ${\text{LAI}}_{j}^{*}$ can be obtained from ${\text{NPP}}_{i}^{*}$ and ${\text{NPP}}_{j}^{*}$ by generalizing equations (A2) and (A5). Substituting and re‐arranging yields two equations, one for ${\text{NPP}}_{i}^{*}$ as a function of NPP_j_ and one for ${\text{NPP}}_{j}^{*}$ as a function of NPP_i_. The intersection of these two equations yields the overall equilibrium values, as shown in Figure A2.

## References

[ece31733-bib-0001] Ainsworth, E. A. , and S. P. Long . 2005 What have we learned from 15 years of free‐air CO_2_ enrichment (FACE)? A meta‐analytic review of the responses of photosynthesis, canopy properties and plant production to rising CO_2_ . New Phytol. 165:351–372. 15720649 10.1111/j.1469-8137.2004.01224.x

[ece31733-bib-0002] Ali, A. A. 2012 Modeling elevated carbon dioxide impacts on plant competition. PhD thesis Macquarie University, Sydney, NSW, Australia.

[ece31733-bib-0003] Ali, A. A. , B. E. Medlyn , K. Y. Crous , and P. B. Reich . 2013 A trait‐based ecosystem model suggests that long‐term responsiveness to rising atmospheric CO_2_ concentration is greater in slow‐growing than fast growing species. Funct. Ecol. 27:1011–1022.

[ece31733-bib-0004] Atkin, O. K. , M. Schortemeyer , N. McFarlane , and J. R. Evans . 1999 The response of fast ‐ and ‐ slow ‐ growing *Acacia* species to elevated CO_2_: analysis of the underlying components of relative growth rate. Oecologia 120:544–554. 10.1007/s004420050889 28308305

[ece31733-bib-0005] Aubier, T.G. 2013 Modeling the effect of rising atmospheric CO_2_ concentration on plant competition. pp. 1–23. Macquarie University, Sydney, NSW, Australia.

[ece31733-bib-0006] Bazzaz, F. A. 1990 The response of natural ecosystems to rising global CO_2_ levels. Annu. Rev. Ecol. Syst. 21:167–196.

[ece31733-bib-0007] Bazzaz, F. A. , and K. Garbutt . 1988 The response of annuals in competitive neighborhoods: effects of elevated CO_2_ . Ecology 69:937–946.

[ece31733-bib-0008] Bazzaz, F. A. , and K. D. M. McConnaughay . 1992 Plant‐plant interactions in elevated CO_2_ environments. Aust. Syst. Bot. 40:547–563.

[ece31733-bib-0009] Berry, S. L. , and M. L. Roderick . 2002 Estimating mixtures of leaf functional types using continental‐scale satellite and climate data. Glob. Ecol. Biogeogr. 11:23–39.

[ece31733-bib-0010] Brooker, R. W. 2006 Plant‐plant interactions and environmental change. New Phytol. 171:271–284. 16866935 10.1111/j.1469-8137.2006.01752.x

[ece31733-bib-0011] Cannell, M. G. R. , and J. H. M. Thornely . 1998 N‐poor ecosystems may respond more to elevated [CO_2_] than N‐rich ones in the long term. A model analysis of grassland. Glob. Chang. Biol. 4:431–442.

[ece31733-bib-0012] Chapin, F. S. III . 1980 The mineral nutrition of wild plants. Annu. Rev. Ecol. Syst. 11:233–260.

[ece31733-bib-0013] Comins, H. N. , and R. E. McMurtrie . 1993 Long‐term response of nutrient‐limited forests to CO_2_ enrichment; equilibrium behaviour of plant‐soil models. Ecol. Appl. 3:666–681. 10.2307/1942099 27759289

[ece31733-bib-0014] Craine, J. M. 2005 Reconciling plant strategy theories of Grime and Tilman. J. Ecol. 93:1041–1052.

[ece31733-bib-0015] Craine, J. M. , J. Fargione , and S. Sugita . 2005 Supply pre‐emption, not concentration reduction, is the mechanism of competition for nutrients. New Phytol. 166:933–940. 15869653 10.1111/j.1469-8137.2005.01386.x

[ece31733-bib-0016] Crous, K. Y. , P. B. Reich , M. D. Hunter , and D. S. Ellsworth . 2010 Maintenance of leaf N controls the photosynthetic CO_2_ response of grassland species exposed to 9 years of free‐air CO_2_ enrichment. Glob. Chang. Biol. 16:2076–2088.

[ece31733-bib-0017] Daufresne, T. , and L. O. Hedin . 2005 Plant coexistence depends on ecosystem nutrient cycles: extension of the resource‐ratio theory. Proc. Natl Acad. Sci. USA 102:9212–9217. 15964989 10.1073/pnas.0406427102 PMC1166585

[ece31733-bib-0018] Dukes, J. S. 2002 Comparison of the effect of elevated CO_2_ on an invasive species (*Centaurea solstitialis*) in monoculture and community settings. Plant Ecol. 160:225–234.

[ece31733-bib-0019] Dybzinski, R. , and D. Tilman . 2007 Resource use patterns predict long‐term outcomes of plant competition for nutrients and light. Am. Nat. 170:305–318. 17879183 10.1086/519857

[ece31733-bib-0020] Farquhar, G. D. , and S. von Caemmerer . 1982 Modelling of photosynthetic response to environmental conditions Pp. 549–587 *in* Lange O. L., Nobel P. S., Osmond C. B. and Ziegler H., eds. Physiological Plant Ecology II. Springer Berlin Heidelberg, Germany.

[ece31733-bib-0021] Grime, J. P. 1979 Plant strategies and vegetation process. John Wiley & Sons, Chicester, UK.

[ece31733-bib-0022] Herbert, D. A. , E. B. Rastetter , G. R. Shaver , and G. I. Agren . 1999 Effects of plant growth characteristics on biogeochemistry and community composition in a changing climate. Ecosystems 2:367–382.

[ece31733-bib-0023] Herbert, D. A. , E. B. Rastetter , L. Gough , and G. R. Shaver . 2004 Species diversity across nutrient gradients: an analysis of resource competition in model ecosystems. Ecosystems 7:296–310.

[ece31733-bib-0024] Hubbell, S. P. 2001 The unified neutral theory of biodiversity and biogeography. Princeton Univ. Press, Princeton, NJ. 10.1016/j.tree.2011.03.024 21561679

[ece31733-bib-0025] Huisman, J. , R. R. Jonker , C. Zonneveld , and F. J. Weissing . 1999 Competition for light between phytoplankton species: experimental tests of mechanistic theory. Ecology 80:211–222.

[ece31733-bib-0026] Huston, M. A. , and D. L. DeAngelis . 1994 Competition and coexistence: the effects of resource transport and supply rates. Am. Nat. 144:954–977.

[ece31733-bib-0027] Isbell, F. , P. B. Reich , D. Tilman , S. E. Hobbie , S. Polasky , and S. Binder . 2013 Nutrient enrichment, biodiversity loss, and consequent declines in ecosystem productivity. Proc. Natl Acad. Sci. USA 110:11911–11916. 23818582 10.1073/pnas.1310880110 PMC3718098

[ece31733-bib-0028] Ju, S. , and D. L. DeAngelis . 2009 The R* rule and energy flux in a plant‐nutrient ecosystem. J. Theor. Biol. 256:326–332. 18977366 10.1016/j.jtbi.2008.10.002

[ece31733-bib-0029] Körner, C. 2003 Ecological impacts of atmospheric CO_2_ enrichment on terrestrial ecosystems. Philos. Trans. A Math. Phys. Eng. Sci. 361:2023–2041. 14558907 10.1098/rsta.2003.1241

[ece31733-bib-0030] Körner, C. , and F. A. Bazzaz . 1996 Carbon dioxide, populations and communities. Academic Press, San Diego, CA.

[ece31733-bib-0031] Lau, J. A. , R. G. Shaw , P. B. Reich , and P. Tiffin . 2010 Species interactions in a changing environment: elevated CO_2_ alters the ecological and potential evolutionary consequences of competition. Evol. Ecol. Res. 12:435–455.

[ece31733-bib-0032] Leadley, P. W. , P. A. Niklaus , R. Stocker , and C. Körner . 1999 A field study of the effects of elevated CO2 on plant biomass and community structure in a calcareous grassland. Oecologia 118:39–49. 20135159 10.1007/s004420050701

[ece31733-bib-0033] Lloyd, J. , and G. D. Farquhar . 1996 The CO_2_ dependence of photosynthesis, plant growth responses to elevated CO_2_ concentrations and their interactions with soil status. I General Principles and forest ecosystems. Funct. Ecol. 10:4–32.

[ece31733-bib-0034] Medlyn, B. E. , R. E. McMurtrie , R. C. Dewar , and M. P. Jeffreys . 2000 Soil processes dominate the long‐term response of forest net primary productivity to increased temperature and atmospheric CO_2_ concentration. Can. J. For. Res. 30:873–888.

[ece31733-bib-0035] Medlyn, B. E. , R. A. E. Duursma , D. A. Ellsworth , I. C. Prentice , C. V. M. Barton , K. Y. Crous , et al. 2011 Reconciling the optimal and empirical approaches to modelling stomatal conductance. Glob. Chang. Biol., 10:1365–2486.

[ece31733-bib-0036] Miki, T. , and M. Kondoh . 2002 Feedbacks between nutrient cycling and vegetation predict plant species coexistence and invasion. Ecol. Lett. 5:624–633.

[ece31733-bib-0037] Moorcroft, P. R. , G. C. Hurtt , and S. W. Pacala . 2001 A method for scaling vegetation dynamics: the ecosystem demography model (ED). Ecol. Monogr. 71:557–586.

[ece31733-bib-0038] Morgan, J. A. , D. E. Pataki , C. Körner , H. Clark , S. J. Del Grosso , J. M. Grünzweig , et al. 2004 Water relations in grassland and desert ecosystems exposed to elevated atmospheric CO_2_ . Oecologia 140:11–25. 15156395 10.1007/s00442-004-1550-2

[ece31733-bib-0039] Newton, P. C. D. , V. Allard , R. A. Carran , and M. Lieffering . 2006 Impacts of elevated CO_2_ on a grassland grazed by sheep: the New Zealand FACE experiment Pp. 157–171 *in* Nörsberger J., Long S., Norby R. J., Stitt M., Hendrey G. R. and Blum H., eds. Managed ecosystems and CO2: case studies, processes, and perspectives. Springer Verlag, Berlin, Germany.

[ece31733-bib-0040] Novotny, A. M. , J. D. Schade , S. E. Hobbie , A. D. Kay , A. D. Kyle , P. B. Reich , et al. 2007 Stoichiometric response of nitrogen‐fixing and non‐fixing dicots to manipulations of CO_2_, nitrogen, and diversity. Oecologia 151:687–696. 17106721 10.1007/s00442-006-0599-5

[ece31733-bib-0041] Oberbauer, S. F. , B. R. Strain , and N. Fetcher . 1985 Effect of CO_2_ ‐ enrichment on seedling, physiology and growth of two tropical tree species. Physiol. Plant. 65:352–356.

[ece31733-bib-0042] Oren, R. , D. S. Ellsworth , K. H. Johnsen , N. G. Phillips , B. E. Ewers , C. Maier , et al. 2001 Soil fertility limits carbon sequestration by forest ecosystems in a CO_2_ ‐ enriched atmosphere. Nature 411:469–472. 11373677 10.1038/35078064

[ece31733-bib-0043] Parton, W. , D. Ojima , and D. Schimel . 1994 Environmental change in grasslands: assessment using models. Clim. Change. 28:111–141.

[ece31733-bib-0044] Passarge, J. , S. Hol , M. Escher , and J. Huisman . 2006 Competition for nutrients and light: stable coexistence, alternative stable states, or competitive exclusion? Ecol. Monogr. 76:57–72.

[ece31733-bib-1000] Polley, H. W. , H. S. Mayeux , H. B. Johnson , and C. R. Tischler . 1997 Viewpoint: Atmospheric CO_2_, soil water, and shrub, grass ratios on rangelands. J. Range. Manage. 50:278‐284.

[ece31733-bib-0045] Poorter, H. 1993 Interspecific variation in the growth response of plants to an elevated ambient CO_2_ concentration. Vegetatio 104:77–97.

[ece31733-bib-0046] Poorter, H. 1998 Do slow ‐ growing species and nutrient ‐ stressed plants respond relatively strongly to elevated CO_2_? Glob. Chang. Biol. 4:693–697.

[ece31733-bib-0047] Poorter, H. , and M. L. Navas . 2003 Plant growth and competition under elevated CO_2_: on winners, losers and functional groups. New Phytol. 157:175–198. 10.1046/j.1469-8137.2003.00680.x 33873640

[ece31733-bib-0048] Potvin, C. , and L. Vasseur . 1997 Long‐term CO_2_ enrichment of a pasture community: species richness, dominance and succession. Ecology 78:666–677.

[ece31733-bib-0049] Rastetter, E. B. , and G. I. Ågren . 2002 Changes in individual allometry can lead to species coexistence without niche separation. Ecosystems 5:789–801.

[ece31733-bib-0050] Reich, P. B. 2009 Elevated CO_2_ reduces losses of plant diversity caused by nitrogen deposition. Science 326:1399–1402. 19965757 10.1126/science.1178820

[ece31733-bib-0051] Reich, P. B. , J. Knops , D. Tilman , J. Craine , D. Ellsworth , M. Tjoelker , et al. 2001a Plant diversity enhances ecosystem responses to elevated CO_2_ and nitrogen deposition. Nature 410:809–812. 11298447 10.1038/35071062

[ece31733-bib-0052] Reich, P. B. , D. Tilman , J. Craine , D. Ellsworth , M. G. Tjoelker , J. Knops , et al. 2001b Do species and functional groups differ in acquisition and use of C, N and water under varying atmospheric CO_2_ and N availability regimes? A field test with 16 grassland species. New Phytol. 150:435–448.

[ece31733-bib-0053] Reich, P. B. , S. E. Hobbie , and T. Lee . 2006a Nitrogen limitation constraints sustainability of ecosystem response to CO_2_ . Nature 440:922–925. 16612381 10.1038/nature04486

[ece31733-bib-0054] Reich, P. B. , B. A. Hungate , and Y. Luo . 2006b Carbon‐nitrogen interactions in terrestrial ecosystems in response to rising atmospheric carbon dioxide. Annu. Rev. Ecol. Syst. 37:611–636.

[ece31733-bib-0055] Reynolds, H. L. 1996 Effects of elevated CO_2_ on plants grown in competition Pp. 273–286 *in* Körner C. and Bazzaz F., eds. Carbon dioxide, populations, and communities. Academic Press, San Diego, CA.

[ece31733-bib-0056] Suding, K. N. , S. L. Collins , L. Gough , C. Clark , E. E. Cleland , K. L. Gross , et al. 2005 Functional‐and abundance‐based mechanisms explain diversity loss due to N fertilization. Proc. Natl Acad. Sci. USA 102:4387–4392. 15755810 10.1073/pnas.0408648102 PMC555488

[ece31733-bib-0057] Thompson, K. 1987 The resource ratio hypothesis and the meaning of competition. Funct. Ecol. 1:297–303.

[ece31733-bib-0058] Tilman, D. 1982 Resource competition and community structure. Priceton Univ. Press, Priceton, NJ. 7162524

[ece31733-bib-0059] Tilman, D. 1997 Community invasibility, recruitment limitation, and grassland biodiversity. Ecology 78:81–92.

[ece31733-bib-0060] Tilman, D. , and D. Wedin . 1991 Dynamics of nitrogen com‐petition between successional grasses. Ecology 72:1038–1049.

[ece31733-bib-0061] Wedin, D. , and D. Tilman . 1993 Competition among grasses along a nitrogen gradient: initial conditions on mechanisms of competition. Ecol. Monogr. 63:199–229.

[ece31733-bib-0062] Woodward, F. I. , and C. K. Kelly . 2008 Responses of global plant diversity capacity to changes in carbon dioxide concentration and climate. Ecol. Lett. 11:1229–1237. 18803643 10.1111/j.1461-0248.2008.01240.x

[ece31733-bib-0063] Wright, I. J. , P. B. Reich , M. Westoby , D. D. Ackerly , Z. Baruch , F. Bongers , et al. 2004 The worldwide leaf economics spectrum. Nature 428:821–827. 15103368 10.1038/nature02403

[ece31733-bib-0064] Zavaleta, E. S. , M. R. Shaw , N. R. Chiariello , H. A. Mooney , and C. B. Field . 2003 Additive effects of simulated climate changes, elevated CO_2,_ and nitrogen deposition on grassland diversity. Proc. Natl Acad. Sci. USA 100:7650–7654. 12810960 10.1073/pnas.0932734100 PMC164642

[ece31733-bib-0065] Zelikova, T. J. , D. M. Blumenthal , D. G. Williams , L. Souza , D. R. LeCain , J. Morgan , et al. 2014 Long‐term exposure to elevated CO2 enhances plant community stability by suppressing dominant plant species in a mixed‐grass prairie. Proc. Natl Acad. Sci. USA 111:15456–15461. 25313034 10.1073/pnas.1414659111 PMC4217402

